# Generation and Characterization of a Polyclonal Human Reference Antibody to Measure Anti-Drug Antibody Titers in Patients with Fabry Disease

**DOI:** 10.3390/ijms22052680

**Published:** 2021-03-06

**Authors:** Malte Lenders, David Scharnetzki, Ali Heidari, Daniele Di Iorio, Seraphine Valeska Wegner, Eva Brand

**Affiliations:** 1 Internal Medicine D, Department of Nephrology, Hypertension and Rheumatology, Interdisciplinary Fabry Center Muenster (IFAZ), University Hospital Muenster, 48149 Muenster, Germany; David.Scharnetzki@ukmuenster.de (D.S.); Eva.Brand@ukmuenster.de (E.B.); 2 Institute of Physiological Chemistry and Pathobiochemistry, University of Muenster, 48149 Muenster, Germany; heidaria@uni-muenster.de (A.H.); diiorio@uni-muenster.de (D.D.I.); wegnerse@uni-muenster.de (S.V.W.)

**Keywords:** Fabry disease, anti-drug antibodies, AGAL, affinities, ELISA

## Abstract

Male patients with Fabry disease (FD) are at high risk for the formation of antibodies to recombinant α-galactosidase A (AGAL), used for enzyme replacement therapy. Due to the rapid disease progression, the identification of patients at risk is highly warranted. However, currently suitable references and standardized protocols for anti-drug antibodies (ADA) determination do not exist. Here we generate a comprehensive patient-derived antibody mixture as a reference, allowing ELISA-based quantification of antibody titers from individual blood samples. Serum samples of 22 male patients with FD and ADAs against AGAL were pooled and purified by immune adsorption. ADA-affinities against agalsidase-α, agalsidase-β and Moss-AGAL were measured by quartz crystal microbalance with dissipation monitoring (QCM-D). AGAL-specific immune adsorption generated a polyclonal ADA mixture showing a concentration-dependent binding and inhibition of AGAL. Titers in raw sera and from purified total IgGs (r^2^ = 0.9063 and r^2^ = 0.8952, both *p* < 0.0001) correlated with the individual inhibitory capacities of ADAs. QCM-D measurements demonstrated comparable affinities of the reference antibody for agalsidase-α, agalsidase-β and Moss-AGAL (KD: 1.94 ± 0.11 µM, 2.46 ± 0.21 µM, and 1.33 ± 0.09 µM, respectively). The reference antibody allows the ELISA-based ADA titer determination and quantification of absolute concentrations. Furthermore, ADAs from patients with FD have comparable affinities to agalsidase-α, agalsidase-β and Moss-AGAL.

## 1. Introduction

Fabry disease (FD, online Mendelian Inheritance in Man (OMIM) no. 301500) is a rare X-chromosomal-linked lysosomal storage disorder, caused by a deficiency of the α-galactosidase A (AGAL; EC 3.2.1.22) enzyme. The progressive accumulation of the AGAL substrate globotriaosylceramide (Gb3) results in a life-threatening multisystemous disease including heart failure, cardiac arrhythmia, cerebrovascular events, and end-stage renal disease. [1] Currently, in addition to chaperone therapy, FD is treatable by enzyme replacement therapy (ERT) using either agalsidase-α (0.2 mg/kg body weight every other week; Shire/Takeda, Lexington, MA, USA) or agalsidase-β (1.0 mg/kg body weight every other week; Sanofi-Genzyme, Cambridge, MA, USA). [2,3] Treatment with either agalsidase-α or agalsidase-β demonstrated beneficial effects on disease progression and manifestations in affected patients. [4] Comparable to other lysosomal storage disorders such as Pompe or Gaucher diseases, which are also treated by ERT, classical male FD patients are at high risk for the formation of persisting neutralizing anti-drug antibodies (ADA) against both components of ERT [5,6,7,8]. Due to the rapid disease progression the measurement of ADA titers is important for an individually tailored treatment management in affected patients. Currently, several different approaches to measure ADAs are used, including ELISA-based measures (also including IgG subclass analyses) [5,9], inhibitory-based measures [5,7,10,11], cell-based measures (to identify effects on cellular ERT uptake) [12] and bed-side tests [13]. All assays are suitable to identify ADAs, but titers are hardly comparable between assays or even between patient cohorts measured by different laboratories. In general, ELISA-based assays are most popular due to the reproducibility and feasibility. However, the weakness of these ELISA-based ADA measures is the lack of an appropriate reference antibody, allowing to quantify the absolute concentration of antibodies in a patients’ sample. Our hypothesis is that a comprehensive patient-derived antibody mixture used as a reference allows an ELISA-based quantification of antibody titers from single blood samples. In contrast to the current applied protocols, this method would allow a simple, fast and reliable determination of antibody concentrations in routine clinical practice.

In the current study, we pooled serum samples from 22 patients with FD and positive for neutralizing ADAs to generate a reference antibody against recombinant AGAL. Subsequently, the reference antibody was used to measure individual ADA titers in 40 ADA-positive FD patients and ELISA-based titers were validated against inhibition-mediated measured titers. Finally, the purified reference antibody was biochemically characterized by measuring the binding affinity to three different recombinant AGALs.

## 2. Results

### 2.1. Generation of an Anti-AGAL Reference Antibody from Human Serum Samples

**Figure 1:**
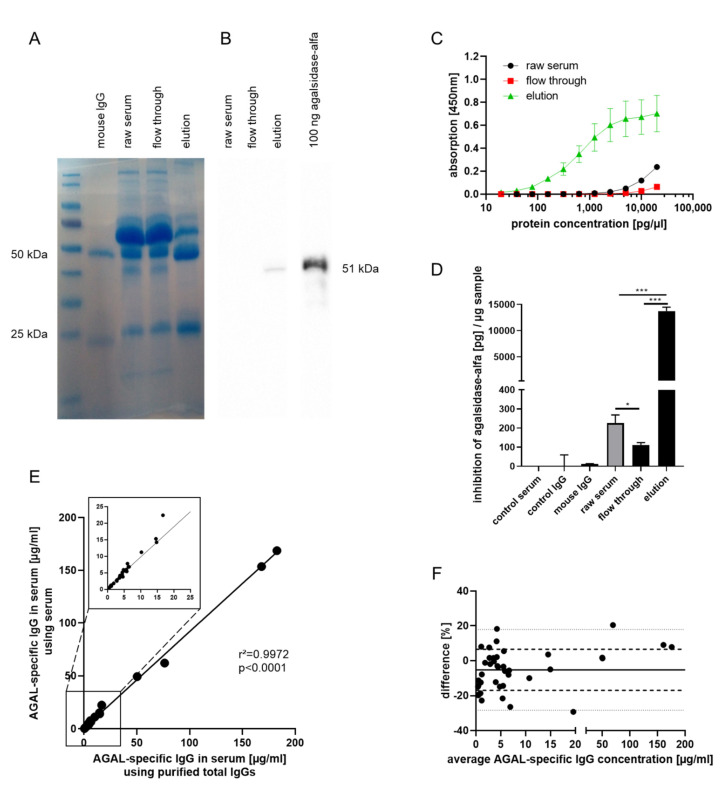
Generation of a human derived reference antibody for ELISA-based determination of anti-AGAL antibody concentrations in patients’ sera. Agalsidase-α-coupled NHS-activated high performance columns were used to extract anti-AGAL antibodies from patients’ sera via immune adsorption. (**A**) SDS-PAGE followed by Coomassie staining showed increased IgG content in the elution fraction indicated by heavy and light chains. (**B**) Western blot analysis with raw serum (before immune adsorption), flow through fraction and elution fraction to detect AGAL. Agalsidase-α was used as positive control. (**C**) ELISA-based detection of AGAL-binding human IgGs using serial dilutions of raw serum fraction, flow through fraction, and elution fraction after immune adsorption (raw serum fraction vs. elution fraction: *p* < 0.0001, flow through fraction vs. elution fraction: *p* < 0.0001). (**D**) Amount of agalsidase-α in pg inhibited by 1 µg of healthy human control serum, purified total IgG from control serum, mouse IgG, raw serum fraction, flow through fraction, and elution fraction after immune adsorption. (**E**) ELISA-based determination of anti-AGAL antibody concentrations in 40 patients’ sera and their corresponding purified total IgGs (**F**) Bland-Altman plot with bias: −5.204% (solid line), SD: −16.974% and 6.566% (dashed lines), 95% limits of agreement: −28.28% and 17.87% (dotted lines). AGAL: α-galactosidase A; IgG: Immunoglobulin G; * *p* < 0.05; *** *p* < 0.001.

The current study aimed to generate an anti-AGAL reference antibody for the direct measure of patients’ anti-AGAL antibody concentrations. Therefore, agalsidase-α-coupled NHS-activated high-performance columns were used to extract anti-AGAL antibodies from 22 AGAL-inhibition positive male patients’ sera by immune adsorption (Figure 1).

**Figure A1:**
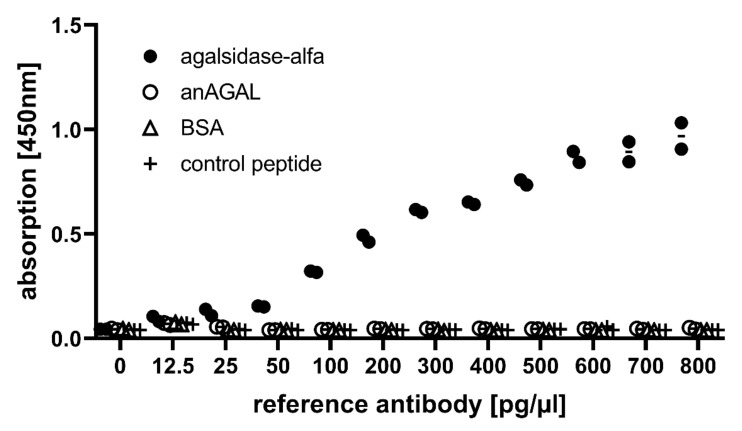
Specificity control of the reference antibody. Increasing antibody concentrations were tested against agalsidase-α, a non-homologous α-galactosidase A from *Aspergillus niger* (anAGAL), bovine serum albumin (BSA) and a control peptide.

To verify a successful immune adsorption, SDS-PAGE with subsequent Coomassie staining and western blot analysis was performed (Figure 1A). Mouse IgG control demonstrated the typical pattern of heavy (50 kDa) and light (25 kDa) IgG chains. IgG level was most dominant in the elution fraction compared to the raw serum and flow through fraction. Western blot analysis was performed to control if agalsidase-α (51 kDa) dissociates from columns during immune adsorption process (Figure 1B). Agalsidase-α was not detectable within raw serum and flow through fraction, however, a slight agalsidase-α signal was observed within the elution fraction. Additional control ELISAs with BSA, a negative control peptide and a non-homologous AGAL from *Aspergillus niger* as baits excluded a non-specific binding of the reference antibody (Figure A1).

Next, ELISA and inhibition assays were performed to determine if IgGs in the elution fraction were specific to AGAL and still have inhibitory capacities. ELISA revealed high concentrations of AGAL-binding IgGs within the elution fraction compared to the raw serum fraction (*p* < 0.0001) and flow through fraction (*p* < 0.0001) (Figure 1C). Inhibition of agalsidase-α by anti-AGAL reference antibody was demonstrated by inhibition assays (Figure 1D). One µg flow through fraction (immune adsorbed serum) (111.6 ± 12.2 pg/µg) showed less inhibition of agalsidase-α than one µg raw serum (225.2 ± 42.9 pg/µg; *p* = 0.0002). The highest inhibitory capacity was observed for the elution fraction (13,737.0 ± 751.4 pg/µg) compared to raw serum (*p* < 0.0001), and flow through fraction (*p* < 0.0001), supporting previous ELISA-based results. Furthermore, these data demonstrate that (in vitro) a 24-fold molar excess of ADAs is required to inhibit the same amount of AGAL.

**Figure A2:**
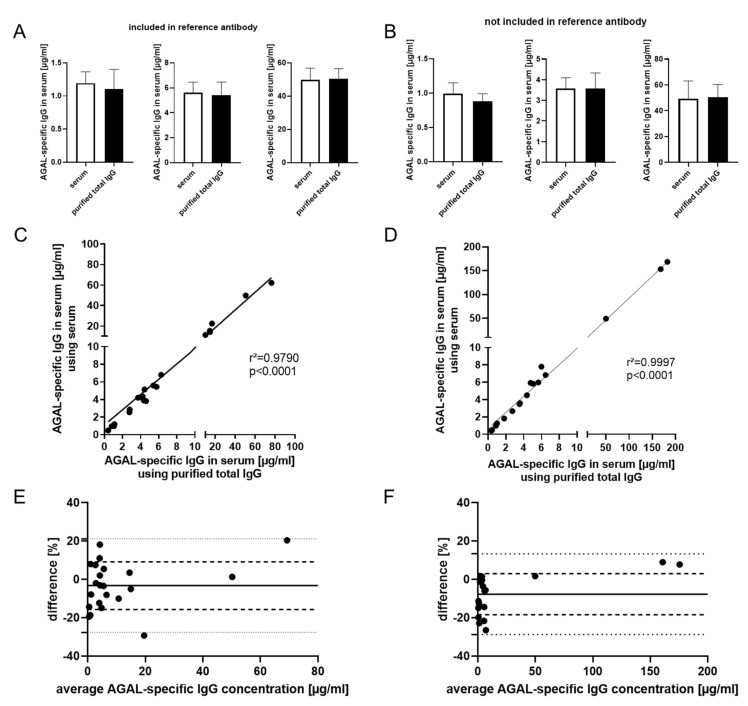
ELISA-based determination of anti-AGAL antibody concentrations. Representative ELISA-based measures using sera and purified total IgGs from patients’ samples (three each), while samples in (**A**) are included and (**B**) are not included in the anti-AGAL reference antibody. The high correlations of anti-AGAL antibody concentration between patients’ sera and their corresponding purified total IgGs in (**C**) reference antibody included (N = 22), respectively, in (**D**) reference antibody not included (N = 18) patients were supported by Bland-Altman plot with (**E**) bias: −3.179% (solid line), SD: −15.619% and 9.261% (dashed lines), 95% limits of agreement: −27.56% and 21.2% (dotted lines) and (**F**) bias: −7.68% (solid line), SD: −18.4% and 3.04% (dashed lines), 95% limits of agreement: −28.7% and 13.34% (dotted lines) AGAL: α-galactosidase A; IgG: Immunoglobulin G.

Raw sera from 40 patients and their corresponding purified total IgGs were used for the ELISA-based determination of anti-AGAL antibody concentrations. Measured concentrations of AGAL-specific IgGs in patients’ sera showed a high correlation between titers from purified total IgGs and sera from different samples (patients) (r^2^ = 0.9972, *p* < 0.0001; Figure 1E). This was confirmed by a Bland-Altman plot showing a low percentage difference between AGAL-specific IgG concentrations measured from patients’ sera or their respective purified total IgGs (Figure 1F). Since 18 from 40 analyzed patients’ sera were not represented in the anti-AGAL reference antibody, individual data (represented and not represented in anti-AGAL reference antibody) are shown in Figure A2. Again, concentrations of AGAL-specific IgGs showed no significant difference between patients’ sera and respective purified total IgGs (Figure A2A,B), resulting in high correlations between samples represented (r^2^ = 0.9790, *p* < 0.0001; Figure A2C) and not represented in anti-AGAL reference antibody (r^2^ = 0.9997, *p* < 0.0001; Figure A2D). This was also supported by Bland-Altman plots showing that almost all data were arranged within 95% confidence interval (−27.56 and 21.2%; Figure A2E, −28.7 and 13.34%; Figure A2F).

### 2.2. Validation of Anti-AGAL Antibody Concentrations in Human Samples

**Figure 2:**
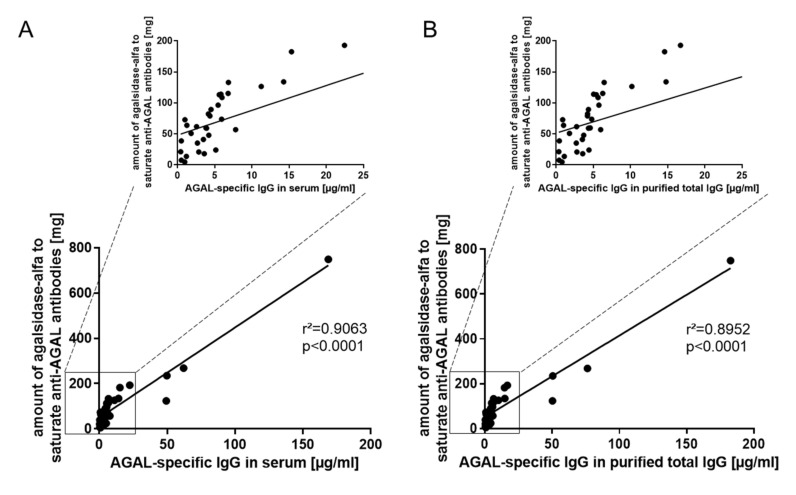
Validation of anti-AGAL antibody concentrations. Amount of agalsidase-α required for antibody saturation was determined for 36 patients using purified total IgGs and plotted against corresponding ELISA-based determined AGAL-specific IgG concentrations determined using (**A**) sera or (**B**) purified total IgGs. AGAL: α-galactosidase A; IgG: Immunoglobulin G.

A validation of ELISA-based measured anti-AGAL antibody concentrations was performed by using titration analyses, previously established in our lab. [8] Therefore, the amount of agalsidase-α required for antibody saturation was measured from 36 patients’ and compared to ELISA-based AGAL-specific IgG concentrations (Figure 2). Patients’ sera (Figure 2A) as well as respective purified total IgGs (Figure 2B) showed high correlations with the inhibitory capacities (r^2^ = 0.9063 and r^2^ = 0.8952, both *p* < 0.0001).

**Figure A3:**
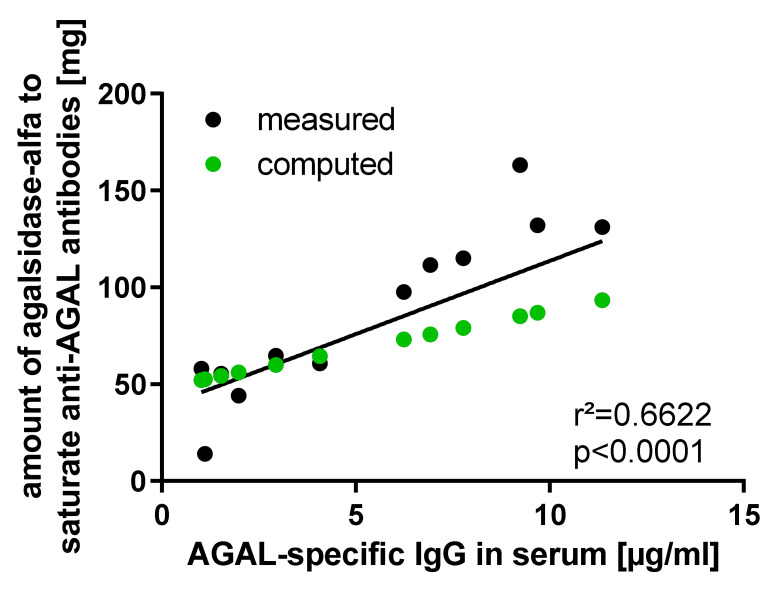
Computation of the amount of agalsidase-α necessary to saturate anti-AGAL antibodies using ELISA-based determination of anti-AGAL antibody concentrations in patients’ sera. ELISA assays were used to determine anti-AGAL antibody concentrations in 12 patients’ sera. Amount of agalsidase-α required for antibody saturation was computed by equation Y = 3.996 × X + 48.10, where X represents AGAL-specific IgG in serum and Y represents amount of agalsidase-α to saturate anti-AGAL-antibodies. AGAL: α-galactosidase A; IgG: Immunoglobulin G.

For a further confirmation, ELISA-based determined anti-AGAL antibody concentrations of 12 patients’ sera were used to compute the amount of agalsidase-α required for antibody saturation. Computed and measured amount of agalsidase-α required for antibody saturation were plotted against corresponding ELISA-based determined anti-AGAL antibody concentrations (r^2^ = 0.6622, *p* < 0.0001; Figure A3).

### 2.3. Biochemical Characterization of the Reference Antibody

**Figure 3:**
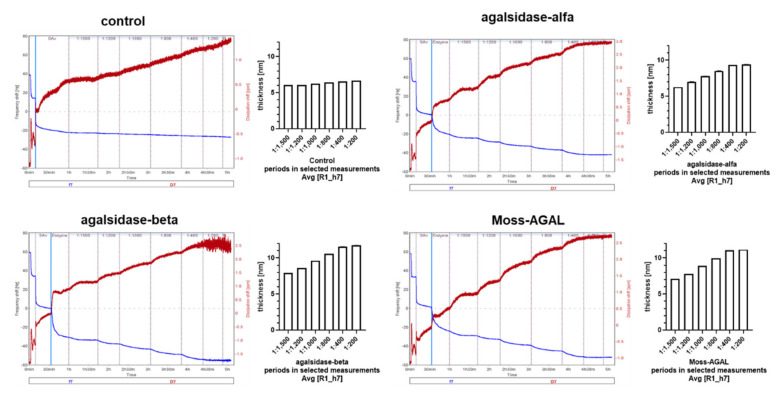
QCM-D-based biochemical characterization of the reference antibody. Control: streptavidin-loaded crystal without AGAL.

**Figure A4:**
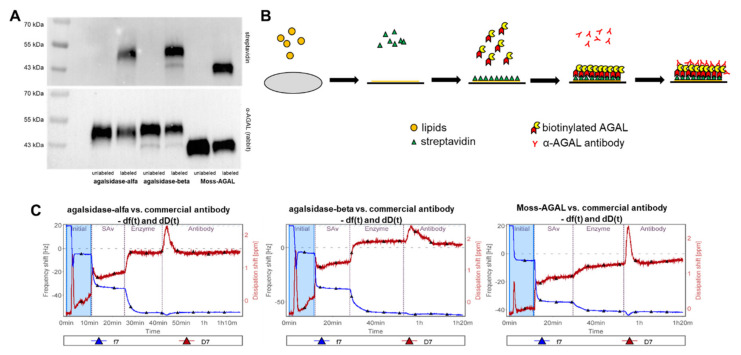
Preliminary experiments for subsequent quartz crystal microbalance with dissipation (QCM-D) analyses. (**A**) Biotin-labelling of agalsidase-α, agalsidase-β and Moss-AGAL. 10 ng respective enzymes (biotin-labeled and unlabeled) were used for western blot analysis. (**B**) Schematic workflow for crystal labelling and measurements. (**C**) QCM-D monitoring to control and measure the binding affinity of a commercial rabbit-derived antibody against agalsidase-α, agalsidase-β, and Moss-AGAL.

Literature showed a cross-reactivity for neutralizing ADAs for agalsidase-α and agalsidase-β [5]. However, to the best of our knowledge, the dissociation constant (KD) of neutralizing ADAs for these enzymes used to treat FD has not been determined so far. In this study, we quantified the interaction between ADAs and different AGALs by quartz crystal microbalance with dissipation monitoring (QCM-D) for the first time. To immobilize AGAL on quartz crystals through streptavidin-biotin interactions, agalsidase-α, agalsidase-β and Moss-AGAL were biotinylated. Western blot analyses demonstrated successful labeling of all three AGALs (Figure A4A) and activity measures revealed no functional disturbances after biotin-labelling (data not shown). Next, the SiO_2_-coated quartz crystals were coated with a lipid bilayer, followed by a streptavidin monolayer (Figure A4B). In QCM-D, the binding of macromolecules to the crystal are detectable as decreases in the frequency and allows for label free and real time monitoring of surface binding events. Subsequently, biotin-labeled AGALs were immobilized on the streptavidin layer on the crystal (Figure A4B). First QCM-D analyses where a commercially available AGAL antibody was passed over the AGAL functionalized crystals, showed significant binding of the antibody to the crystals and allows detecting of all three different AGALs (Figure A4C). Subsequently, different concentrations of the anti-AGAL reference antibody were passed over agalsidase-α, agalsidase-β and Moss-AGAL functionalized crystals, demonstrating a concentration-dependent binding compared to control (without biotin-coupled AGAL; Figure 3). The adsorbed mass was calculated from the changes in frequency using the Sauerbrey equation (Figure 3). Subsequently, the concentration dependent binding of the ADAs to the crystals was used to compute the dissociation constants and revealed comparable binding to agalsidase-α and agalsidase-β (KD: 1.94 ± 0.11 µM and 2.46 ± 0.21 µM, respectively) as well as for Moss-AGAL (KD: 1.33 ± 0.09 µM).

## 3. Discussion

The formation of neutralizing ADAs against infused AGAL has a major impact on therapy efficiency and thus disease progression in affected male patients with FD [7,8]. Therefore, it is highly warranted to identify patients at risk and quantify ADA titers for subsequent individual therapeutic approaches. In the current study, we provide a method to generate a polyclonal reference antibody from human serum samples for ELISA-based ADA titer measurements.

Antibody titers from different studies and even from the same cohorts are usually difficult to compare. This problem is also well known in other LSDs and the reasons are multifactorial. In absence of a reference antibody, ELISA-based assays for antibody measurement are usually expressed as relative values compared to either an ERT-naïve sample (best case) or, if no ERT-naïve sample is available (most commonly), a negative control sample. Furthermore, commercially available antibodies recognize only a limited number of epitopes, which not necessarily represent the antibody of interest, and are from other host species, requiring own secondary antibodies for detection compared to the human samples. Our method, using a polyclonal reference antibody from human samples allowed the measurement of ADAs, reflecting the real antibody concentration (µg/mL) of the affected patients. After purification, the reference antibody still demonstrated an inhibitory capacity against AGAL. However, as recently demonstrated, ADAs from affected patients with FD can also bind to other, non-catalytically important domains for example with direct effects on cellular uptake [12]. Since a 24-fold molar excess of the reference antibody was required to inhibit AGAL, it can be concluded that in addition to enzyme activity neutralizing ADAs also non-inhibitory antibodies against AGAL were purified. However, no conclusions should be drawn to the general inhibitory capacities of individual ADAs in patients since some of the antibodies with inhibitory effects might be missed during the purification due to weaker binding affinities. Thus, although an ELISA-based measure might be superior to detect all free ADAs in affected patients compared to an inhibition assay, for a comprehensive determination of the ADA status functional assays should be performed in affected patient, too. Our ELISA data were supported in that the individual titers correlated well with the amount of enzyme required for ADA saturation during infusions [8]. In this respect, since ADAs can be saturated by AGAL during infusions [11], serum samples for ADA titer measures should be drawn ideally directly before the next infusion, or at least one week after an infusion to minimize false negative results.

ADAs from patients with FD demonstrate a high cross-reactivity against agalsidase-α as well as agalsidase-β [5,7]. However, to the best of our knowledge, the affinities (KD) for different AGALs is unknown so far. Our QCM-D analysis shows that the polyclonal reference antibody has comparable KDs against agalsidase-α and agalsidase-β, as well as against Moss-AGAL. Moss-AGAL (ELEVA) is a recombinant human AGAL expressed in the genetically modified moss *Physcomitrella patens*. Preclinical studies suggest an improved uptake of the enzyme by mannose receptors instead of mannose-6-phosphate receptors into cells [14], while data from a phase I study showed good safety and tolerability of Moss-AGAL after a single dose of 0.2 mg/kg i.v. [15]. Although we observed slight differences for KDs against agalsidase-α, agalsidase-β and Moss-AGAL, KDs were in the same magnitude (10^−6^ M). In addition, it can be concluded that the plant-based production of AGAL does not result in an increased affinity of present ADAs.

High ADA affinities seem to be associated with increasing inhibitory capacities [16], while decreasing affinities might show a beginning of tolerization [17]. Hence, future research determining individual ADA affinities from affected FD patients against the infused enzyme is now warranted to assess if individual differences might occur and if they affect treatment outcomes by reduced AGAL inhibition, while decreasing affinities over longitudinal measures might show a beginning of tolerization of the patient. Therapeutic options or conditions lowering ADA affinities could also be an aim of future research.

## 4. Materials and Methods

### 4.1. Patients’ Samples

Adult male FD patients (*n* = 40) with at least 6 months of ERT (agalsidase-α or agalsidase-β) and positive for neutralizing ADAs were included. Presence of neutralizing ADAs was determined and measured routinely in our lab using serum-mediated inhibition assays [5,6,7]. Time point of serum collection and determination of neutralizing ADA status was the last visit (2016–2020). Serum samples for the reference antibody or for individual ADA measures were drawn at least one week after the last infusion. Only one sample from this visit was used for the generation of the reference antibody and for subsequent ADA titer measures. Previous reports demonstrated a significant variation of antibody epitopes against AGAL. [12,18] Therefore, patients’ samples with known ADA epitopes were used [18] to ensure that the reference antibody represents a wide spectrum of antibody epitopes against AGAL.

### 4.2. Purification of Total IgGs from Human Sera

Total IgGs from patients’ sera for titer measures were purified by negative selection as described previously using Melon Gel IgG Spin Purification Kit (Thermo Fisher Scientific, Darmstadt, Germany) according to manufacturer’s instructions [8,11]. In brief, 100 µL serum were diluted 1:10 with Melon Gel buffer, incubated with 100 µL settled Melon Gel, and inverted for 5 min at room temperature. After protein adsorption, total IgGs were separated via centrifugation at 12,000× *g* for 5 min. BCA (Thermo Fisher Scientific) and SDS-PAGE analysis was performed as reported previously to estimate the purified IgG content and to control the success of purification [11].

### 4.3. Generation of a Reference Antibody by Immune Adsorption

To adsorb and purify anti-AGAL antibodies from patients’ sera by positive selection, the immune adsorption was performed as described previously. [12] In short, 1 mg agalsidase-α (Shire/Takeda) was coupled to 1 mL HiTrap N-hydroxysuccinimide (NHS)-activated high performance columns (GE Healthcare, Freiburg, Germany; no. 17071601) according to the manufacturer’s instructions. After ligand coupling, columns were washed, deactivated and equilibrated. Sera from 22 patients (each 100 µL) were pooled, diluted 1:10 with 1× PBS and loaded on the column. The column was washed with eight column volumes 1× PBS and the flow through fraction containing the unbound serum proteins was collected for later analysis. Anti-AGAL antibodies were eluted with 100 mM glycine pH 2.2 and directly neutralized with 1 M Tris-HCl pH 9. Elution fractions were concentrated, dialyzed and collected in 1× PBS to receive the anti-AGAL reference antibody. Raw serum fraction, flow through fraction, and elution fraction were used for SDS-PAGE analysis followed by Coomassie staining and western blot analysis to verify successful immune adsorption. To further characterize the reference antibody, ELISA and inhibition assays were performed.

### 4.4. SDS-Page and Western Blot Analysis

To detect IgGs within raw serum, flow through, and elution fractions, SDS-Page was performed. In short, 15 µg samples were used for SDS-Page followed by subsequent Coomassie staining. Coomassie staining was performed according to manufacturer’s instructions (Thermo Fisher Scientific). Mouse IgG was loaded as positive control. For western blot analysis 10 µg samples and 100 ng agalsidase-α as positive control were blotted onto PVDF membranes. After blocking overnight in Tris buffered saline with 5% milk powder, detection was performed using an anti-AGAL antibody (ab168341, Abcam, Cambridge, UK; working concentration: 100 ng/mL) and a secondary horseradish-peroxidase-labeled goat anti-rabbit IgG antibody (12–348, Sigma-Aldrich, St. Louis, MO, USA; working concentration: 100 ng/mL).

### 4.5. ELISA-Based Measurement of AGAL-Binding IgGs

96-well plates were coated with 100 ng agalsidase-α per well over night at 4 °C and washed three times with PBS. For negative controls, 100 ng BSA per well was used. After blocking with 2% BSA/PBS for 1 h at room temperature, wells were washed again. To detect extracted anti-AGAL antibodies from patients’ sera by immune adsorption, serial dilutions of elution, flow through, and raw serum were loaded into the wells and incubated for 2 h at room temperature. After five washing steps with 0.1% Tween-20/PBS, anti-hIgG antibodies conjugated with HRP (ab98624, Abcam; working concentration: 20 ng/mL) were applied and incubated for 1 h at room temperature. Wells were washed again five times with 0.1% Tween-20/PBS. For IgG detection 50 µL 1-Step TMB-ELISA Substrate Solution (Thermo Fisher Scientific) were added to the wells, followed by 50 µL 2 M sulfuric acid to stop the reaction after 15 to 20 min. Absorption was measured at 450 nm.

To measure ADA titers from patients’ sera, serial dilutions of 40 (22 represented in anti-AGAL reference antibody and 18 additional) sera and corresponding purified total IgGs, both starting with 4 µL in 100 µL PBS, were loaded into the wells and incubated for 2 h at room temperature. To ensure that patients’ sera and purified total IgGs had the same IgG and protein concentration, sera were diluted 1:10 with Melon Gel buffer and purified total IgGs were supplied with an individual amount of BSA before loading into the wells. A serial dilution of the anti-AGAL reference antibody, starting with 800 pg/µL, was used as reference. IgG detection was performed as described above. To calculate AGAL binding IgG concentrations, linear regressions within serial dilutions of patients’ sera and purified total IgGs were applied. Finally, the concentrations were calculated using the equation obtained from the anti-AGAL antibody reference curve.

To exclude a non-specific binding of the reference antibody, individual ELISAs with 100 ng per well of agalsidase-α, BSA, a negative control peptide (sequence: HWYITTGPVREK) [12,18], and a non-homologous AGAL from *Aspergillus niger* [19] were performed.

### 4.6. Biotin-Labelling of Commercial AGAL

For subsequent QCM-D measurements, agalsidase-α, agalsidase-β and Moss-AGAL (each 1 mg) were biotinylated using the EZ-Link™ Sulfo-NHS-LC-Biotinylation Kit (21435, Thermo Fisher Scientific) according to the manufacturer’s instructions. The calculated coupling was 1 to 2 biotin molecules per AGAL molecule. Biotinylation was controlled by western blot analysis using HRP-coupled streptavidin to detect biotinylated AGAL.

### 4.7. Preparation of Small Unilamellar Vesicles (SUVs)

SUVs were prepared as reported previously [20,21]. Lipids were first dissolved in chloroform and mixed in the desired molar ratio in a glass vial (25 mg/mL 1,2-dioleoyl-sn-glycero-3-phosphocholine (DOPC) and 2 mol% 1,2-dioleoyl-sn-glycero-3-phosphoethanolamine-N-(biotinyl) (DOPE-biotin)). Subsequently, the solvent was evaporated with a nitrogen stream while simultaneously turning the vial in order to obtain a lipidic film. The residing solvent was removed for at least 1 h in a desiccator connected to a vacuum pump. The dried film was re-hydrated in MilliQ water to a concentration of 1 mg/mL, vortexed to ensure that the lipids were fully dissolved and transferred into an Eppendorf tube. The lipids were sonicated for about 30 min until the opaque solution turned clear right before use. The obtained SUVs were stored in the fridge and used within two weeks.

### 4.8. QCM-D Measurements

QCM-D measurements were performed with a Qsense Analyser from Biolin Scientific using SiO_2_-coated sensors (QSX303, Biolin Scientific, Gothenburg, Sweden). Measurements were performed at 22 °C using four parallel flow chambers and one Ismatec (Grevenbroich, Germany) peristaltic pump with a flow rate of 75 µL/min. In this work, the seventh overtone was used for the normalized frequency (Δf7) and dissipation (ΔD7). QSense Dfind software from Biolin Scientific and the standard Sauerbrey modeling was used to calculate the film thickness. QCM-D sensors were first cleaned by immersion in a 2 wt% sodium dodecyl sulfate solution for 30 min and subsequently rinsed three times with Milli-Q water and then with ethanol. The sensors were then dried under a nitrogen stream and activated with 10 min UV/ozone treatment using a UV/ozone cleaner (Ossila, Sheffield, United Kingdom).

For the formation of supported lipid bilayers (SLBs), small unilamellar vesicles (SUVs) were diluted to a concentration of 0.1 mg/mL in buffer solution (50 mM Tris, 100 mM NaCl, pH 7.4) containing 10 mM of CaCl_2_ directly before use and flushed into the chambers after obtaining a stable baseline. The quality of the SLBs was monitored in situ, where high quality SLBs are defined by Δf = −24 ± 1 Hz and ΔD < 0.5 × 10^−6^. Afterwards, a solution of streptavidin SAv (3 μM) was passed over the SLB and followed by the addition of enzymes (agalsidase-α, agalsidase-β, or Moss-AGAL (2 ng/mL, each). Each solution was incubated on the QCM-D crystal until a stable plateau was reached and was subsequently rinsed away with buffer. For the titrations, dilutions of antibodies were passed over the QCM-D crystals ranging from 1:1,500 to 1:200 dilution of a stock solution (1 µg/mL).

### 4.9. Inhibition Assay and Titration of Neutralizing ADAs

To further control if AGAL inhibiting antibodies were extracted from patients’ sera by immune adsorption, inhibition assays were performed as described previously [8,11]. In short, 1 µg flow through fraction, raw serum, and negative controls (mouse IgG, serum from a healthy control, and purified total IgGs from the healthy control) were pre-incubated with 1 ng agalsidase-α for 10 min at room temperature. To calculate the inhibitory capacity of the elution fraction, 100 ng of the reference antibody were pre-incubated with increasing amounts of agalsidase-α (0 to 20 ng) [8]. Residual AGAL activity was determined using 4-methylumbelliferyl-α-D-galactopyranoside (Biosynth, Staad, Switzerland). N-acetylgalactosamine (Santa Cruz Biotechnology, Dallas, TX, USA) was used to inhibit endogenous α-galactosidase B activity [22]. Finally, the amount of inhibited agalsidase-α was calculated (pg per µg sample).

The amount of agalsidase-α required to saturate ADAs in patients’ sera was determined as described previously [8]. In short, 5 µg patients’ purified total IgGs were pre-incubated with a serial dilution of agalsidase-α for 10 min at room temperature. To express agalsidase-α inhibition in percent, residual AGAL activities were normalized against inhibition-negative controls. Agalsidase-α inhibition was plotted against the amount of agalsidase-α and saturation was defined as the amount of enzyme required to reduce the neutralizing capacity of 5 µg patients’ total IgG below the ERT neutralizing threshold of 10% (background threshold) [8,11].

### 4.10. ELISA-Based Calculation of the Amount of Agalsidase-α to Saturate Anti-AGAL-Antibodies

ELISA-based determined anti-AGAL antibody concentrations were used to estimate the amount of agalsidase-α to saturate anti-AGAL-antibodies. Correlation of 36 patients’ anti-AGAL antibody concentrations determined in serum and their corresponding measured amount of agalsidase-α required for antibody saturation resulted in the equation Y = 3.996 × X + 48.10, where X is the AGAL-specific IgG concentration in serum (µg/µL) and Y is the amount of agalsidase-α to saturate anti-AGAL antibodies (mg).

### 4.11. Statistics

If not stated otherwise, all experiments were performed at least three times. Continuous variables were expressed as mean with standard deviation (SD). Two-tailed student’s t test, one-way analysis of variance (ANOVA) with correction for multiple testing or two-way ANOVA with Tukey test were used for statistical analysis. Correlation analyses were performed using Pearson correlation coefficient (r^2^). *p*-values < 0.05 were considered as statistically significant. For appropriate statistical analyses and visualization GraphPad PRISM v8.0 software (GraphPad Software Inc., La Jolla, CA, USA) was used.

## 5. Conclusions

We conclude that the generation of a reference antibody from human blood samples is a feasible tool for ELISA-based ADA titer determination, allowing to express titers as real concentrations. Furthermore, ADAs from patients with FD have comparable affinities to agalsidase-α and agalsidase-β, as well as Moss-AGAL.

## Data Availability

All data and material are present within the main manuscript.
